# Accuracy of structure-based sequence alignment of automatic methods

**DOI:** 10.1186/1471-2105-8-355

**Published:** 2007-09-20

**Authors:** Changhoon Kim, Byungkook Lee

**Affiliations:** 1 Laboratory of Molecular Biology, Center for Cancer Research, National Cancer Institute National Institutes of Health, Bethesda, Maryland, USA

## Abstract

**Background:**

Accurate sequence alignments are essential for homology searches and for building three-dimensional structural models of proteins. Since structure is better conserved than sequence, structure alignments have been used to guide sequence alignments and are commonly used as the gold standard for sequence alignment evaluation. Nonetheless, as far as we know, there is no report of a systematic evaluation of pairwise structure alignment programs in terms of the sequence alignment accuracy.

**Results:**

In this study, we evaluate CE, DaliLite, FAST, LOCK2, MATRAS, SHEBA and VAST in terms of the accuracy of the sequence alignments they produce, using sequence alignments from NCBI's human-curated Conserved Domain Database (CDD) as the standard of truth. We find that 4 to 9% of the residues on average are either not aligned or aligned with more than 8 residues of shift error and that an additional 6 to 14% of residues on average are misaligned by 1–8 residues, depending on the program and the data set used. The fraction of correctly aligned residues generally decreases as the sequence similarity decreases or as the RMSD between the C_*α *_positions of the two structures increases. It varies significantly across CDD superfamilies whether shift error is allowed or not. Also, alignments with different shift errors occur between proteins within the same CDD superfamily, leading to inconsistent alignments between superfamily members. In general, residue pairs that are more than 3.0 Å apart in the reference alignment are heavily (>= 25% on average) misaligned in the test alignments. In addition, each method shows a different pattern of relative weaknesses for different SCOP classes. CE gives relatively poor results for *β*-sheet-containing structures (all-*β*, *α*/*β*, and *α*+*β *classes), DaliLite for "others" class where all but the major four classes are combined, and LOCK2 and VAST for all-*β *and "others" classes.

**Conclusion:**

When the sequence similarity is low, structure-based methods produce better sequence alignments than by using sequence similarities alone. However, current structure-based methods still mis-align 11–19% of the conserved core residues when compared to the human-curated CDD alignments. The alignment quality of each program depends on the protein structural type and similarity, with DaliLite showing the most agreement with CDD on average.

## Background

Accurate sequence alignments for homologous proteins are essential for constructing accurate motifs and profiles, which are used in motif- or profile-based protein function search models [1-3] and in building homology models[4,5]. When sequence similarity is low, however, it is difficult to obtain the correct sequence alignment based on sequence similarity alone [3,4]. Since it is well known that proteins can have similar structures even in the absence of any detectable sequence similarity, structural alignments have been used to guide sequence alignments and are used as the gold standard for sequence alignment evaluation [5,6].

Many pairwise structure alignment programs have been developed, but their performance has often been measured by how well the programs reproduce an expert-curated structure classification, such as SCOP or CATH [7,8]. It has been shown that some programs do not produce high quality individual alignments, as measured by geometric match measures such as SAS or GSAS, even when they perform well in classification tests [9]. It is also known that structure-based sequence alignments produced by different programs can be different even when the superimposed structures are similar [4,5,10-12]. Nonetheless, as far as we know, there is no report of a systematic evaluation of commonly used structural alignment programs in terms of the sequence alignment accuracy, perhaps because it has been difficult to find a fully human-curated and reasonably difficult reference alignment set [13,14].

There are a number of sequence alignment databases that are augmented by structural alignments, including CAMPASS[15], HOMSTRAD[16], PALI[17,18], DBAli[19], PASS2[20], CDD[21], SUPFAM[22], BAliBase[19], OXBench[23], PREFAB[24], SABmark[25] and S4[13]. The extent of similarity of the structures in these databases varies and so does the degree with which the alignments were curated by human experts after they were initially generated by automatic methods and/or imported from outside sources.

Zhu and Weng[26] used HOMSTRAD database to measure the performance of their structure alignment program, FAST, and reported an average accuracy of 96%, measured as the percentage of correctly aligned residues among all aligned residues in the reference alignment. But our study reported herein indicates that such high accuracy is generally not obtained unless the structures are highly similar.

In this study, we evaluate the accuracy of structure-based sequence alignments produced by seven pairwise structure alignment programs, using the human-curated sequence alignments from NCBI's CDD [21] as the standard of truth. This is an expert-curated database, built by importing sequence alignments from outside sources, which are manually modified by considering structure-based alignments. In addition to the family-level alignments, where protein sequences are highly similar, it also provides fully curated superfamily-level alignments, where sequence similarity is not so high[21].

## Results

### Average performance of each method

**Figure 1:**
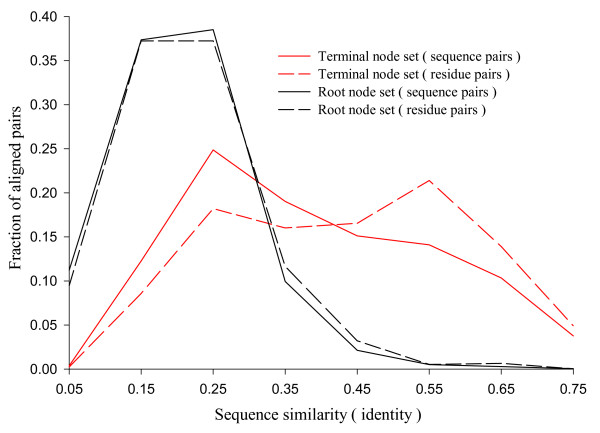
**Distribution of reference alignments over sequence similarity**. The sequence similarity is the fraction of identical residue pairs among all aligned pairs. The fraction of sequence pairs (solid lines) and residues pairs (dashed lines) are plotted in each range of sequence similarities for the root (black) and the terminal (red) node sets. The terminal node set includes 2,199 alignments and 288,401 aligned residue pairs. The root node set includes 4,017 alignments and 245,817 aligned residue pairs. The x-axis gives the mid-point of the similarity range bins of size 0.1. The distribution of the residue pairs is slightly shifted to the right compared to that of the sequence pairs. This implies that there are some large structures with high sequence similarity.

We prepared two reference alignment sets from CDD database as described in the Methods section: the root node set and the terminal node set. Figure 1 shows the distribution of alignments in these reference sets according to the sequence similarity (sequence identity among the aligned residue pairs). The alignments in the terminal node set are distributed over the entire similarity range. The root node set shows narrower distribution, with the peak at about 20% of sequence identity. Both cover all four major SCOP classes (Table 1).

**Table 1:** The composition of the reference alignment datasets

SCOP class	Root node set		Terminal node set	
	
	CDs	Pairs	CDs	Pairs
all-*α*	11	326	37	763
all-*β*	16	1798	35	544
*α*/*β*	36	1203	106	621
*α*+*β*	28	565	55	204
others^†^	10	125	16	67
total	101	4017	249	2199

^† ^Other than the four major classes.

We use "correctly" aligned fraction of residues (*f*_*car*_) as a measure of alignment quality. This measure is defined as the ratio of the number of residues that are aligned correctly, within a specified shift error, to the total number of aligned residues in the reference alignment (see Methods section for details). Since there is a large variation in the number of alignments in the CD nodes, (e.g., 1424 pairs for the immunoglobulin root node cd00096 vs. one pair in the root node cd00120), we use the node-wide average of *f*_*car*_, which we denote as F_car_. In order to compare the performance of different structure-alignment programs, we take the average of F_car _(double average of *f*_car_) over all nodes within each node set.

**Figure 2:**
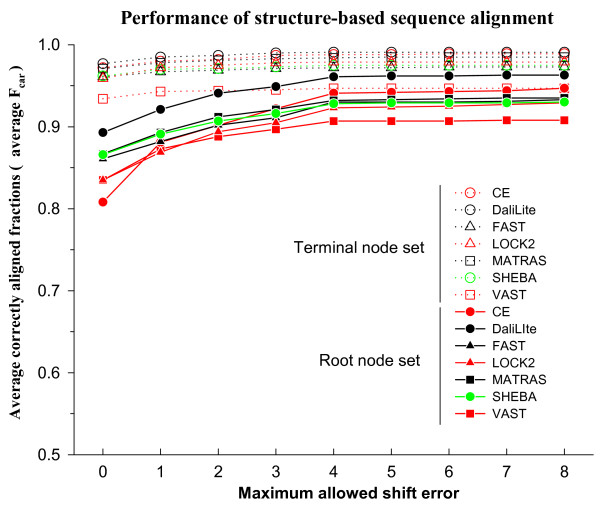
**Average F_car _as a function of the magnitude of the allowed shift error**. The terminal and the root node sets are indicated by dotted lines with open symbols and solid lines with closed symbols, respectively. Program names are given in alphabetical order. Note that the y-axis scale is from 0.5 to 1.0.

With the terminal node set, the different structure comparison methods correctly aligned 93% to 97% of the residues, on average, without shift error (Figure 2). As the allowed shift error is increased up to 8 residues, the F_car _value increases by about 2% for all methods. Thus all methods work well for this dataset. We used the root, rather than the terminal, node set in the analysis of the results reported below. The results with the terminal node set are given in the supplementary material (See Additional file 1). We will often refer to each root node as a superfamily.

In contrast to the terminal node set, the F_car _values without shift error are only 0.81 to 0.89 for the root node set. About 6% to 14% of the residues, on average, are aligned with some shift error (at most 4 residues in general) and an additional 4% to 9% of the residues are either not aligned or aligned with shift error of more than 8 residues. The best performance was achieved by DaliLite, whether shift error was allowed or not. CE was the most dependent on allowed shift error; it ranked the lowest when shift error was not allowed but the second best, after only DaliLite, if a shift error of up to 4 was allowed.

Figure 2 also shows that the average F_car _value changes noticeably between shift error of 0 and 4 but that it remains essentially unchanged after 4. For accurate profile construction, one cannot tolerate a shift error of any magnitude. On the other hand, for the purpose of recognizing similar structures in the database, precise accuracy of the alignment is of less concern. Therefore, we generally focus on *f*_car _values with shift errors of either 0 or up to 8 in the following analysis of the results of this study.

**Figure 3:**
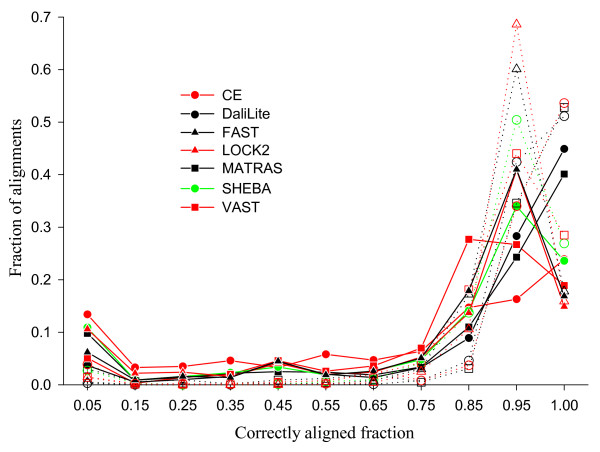
**Distribution of *f*_car _values in the root node set**. The fraction of sequence pairs is plotted in each range of *f*_car _values for *δ *= 0 (solid lines with closed symbols) and 8 (dotted lines with open symbols). The x-axis gives the mid-point of the *f*_car _range bins of size 0.1. The last bin includes only the sequence pairs with *f*_car _= 1.0.

Figure 3 shows the distribution of shift errors among different alignments. When shift errors of up to 8 are allowed (dotted lines), most alignments have *f*_car_(8) values between 0.8 and 1.0 with a peak at 0.95. Even for the worst performer, only about 4% of the alignments are nearly complete failures (the first bin; see also Additional file 2). On the other hand, when no shift error is allowed (solid lines), the correctly aligned fraction is more broadly distributed, with a significant number of alignments in which almost all residues are aligned incorrectly. This is the expected pattern if, in many alignments, all or part of the structure is shifted together by a few residues compared to the reference alignment.

**Figure 4:**
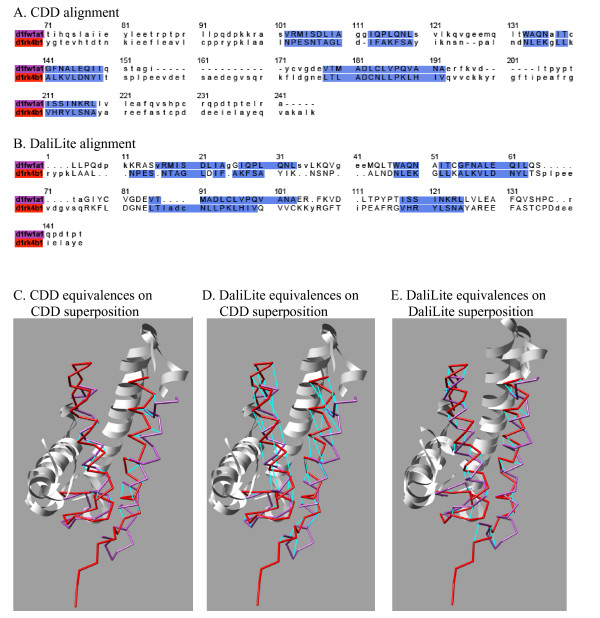
**The comparison of CDD and DaliLite alignments for an all-*α *protein pair from the superfamily cd00299**. The structure-based sequence alignment produced by CDD (A) and DaliLite (B) for two *α*-helical proteins. The color in the sequence name is used for the corresponding structure in the structure superpositions below. The aligned residues are indicated by the upper case letters. The residues aligned in the reference alignment are shaded blue. These sequence alignments were used to generate structural superpositions, by CDD in the left and middle panels (C and D) and by DaliLite in the right panel (E). The orientation of the red structure (d1neu_) is the same in all three panels. Aligned residue pairs are connected by cyan lines, in the left panel according to the CDD and in the middle and right panels according to the DaliLite alignments. Short fragments at the C-termini were cut off and the regions where CDD and DaliLite agree are shown in ribbon, for better visibility of the equivalences. DaliLite achieved 0.453, 0.953 and 0.953 for *f*_car_(0), *f*_car_(4) and *f*_car_(8), respectively. The pictures were prepared using CHIMERA (UCSF, Computer Graphics Lab).

**Figure 5:**
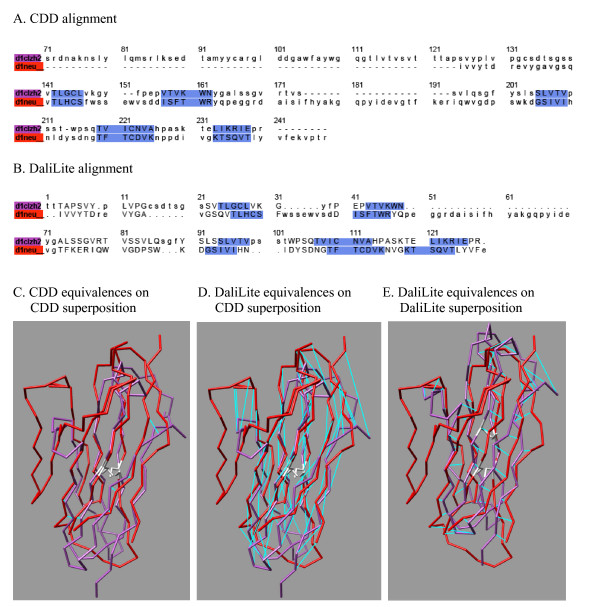
**The comparison of CDD and DaliLite alignments for an all-*β *protein pair from the superfamily cd00096**. The structure-based sequence alignment produced by CDD (A) and DailLite (B) for two immunoglobulin proteins. The conserved cysteine pairs are colored in white. Otherwise, the same as in Figure 4. For this pair, all methods but VAST agreed with DaliLite, while VAST agreed with CDD. DaliLite achieved 0.0, 1.0 and 1.0 for *f*_car_(0), *f*_car_(4) and *f*_car_(8), respectively.

A couple of examples of such shifted alignments are shown in Figures 4 and 5. The former shows an *α*-helical protein pair from cd00299, for which the DaliLite alignment has two of the four helices shifted by one helix turn compared to the CDD alignment. The latter is a pair of immunoglobulin folds from cd00096, for which the DaliLite alignment is shifted by one beta-strand pitch for the entire structure.

### Dependence of performance on sequence similarity and distance between homologous residues

**Figure 6:**
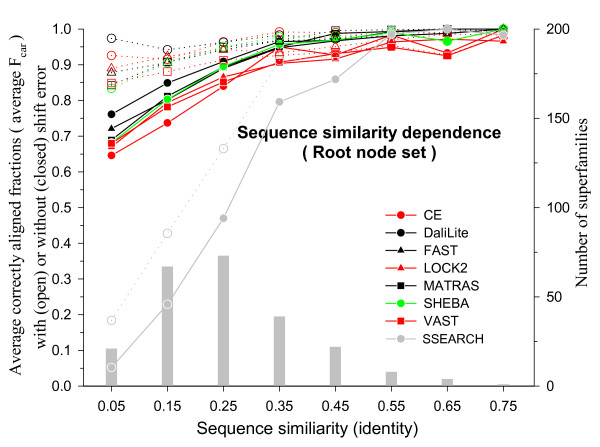
**Sequence similarity (fraction of identical pairs) dependence of F_car _in the root node set**. Alignments were grouped into sequence similarity bins of size 0.1 and then the alignments within each bin were grouped according to its CD name for averaging. The avearge F_car _values are shown with the scale on the left y-axis: open symbols, F_car_(8); closed symbols, F_car_(0). The x-axis shows the midpoint of each sequence similarity bin. The histogram (grey bars) shows the number of superfamilies in each bin with the scale on the right y-axis.

It is reasonable to expect that the alignment accuracy depends on the degree of similarity of the two structures compared. Since proteins with high sequence similarity tend to be structurally similar, the alignment accuracy is expected to depend also on the sequence similarity. Figure 6 shows the average F_car_(0) and F_car_(8) values in different sequence similarity ranges for different methods. As expected, both measures of alignment accuracy fall as the sequence similarity decreases for most methods. Different methods perform similarly well when the sequence similarity is high but their differences become more apparent at the low sequence similarity ranges. DaliLite gives the best average F_car _values. At the low sequence similarity ranges (below 30% identity), CE gives the worst average F_car_(0) values, but the second best average F_car_(8). We have included in Figure 6 for comparison the alignment accuracy obtained by SSEARCH [27], which is a pure sequence alignment procedure. Not surprisingly, all structure-based alignment methods perform much better than the pure sequence alignment method unless the sequence similarity is very high (≥ 50% identity).

**Figure 7:**
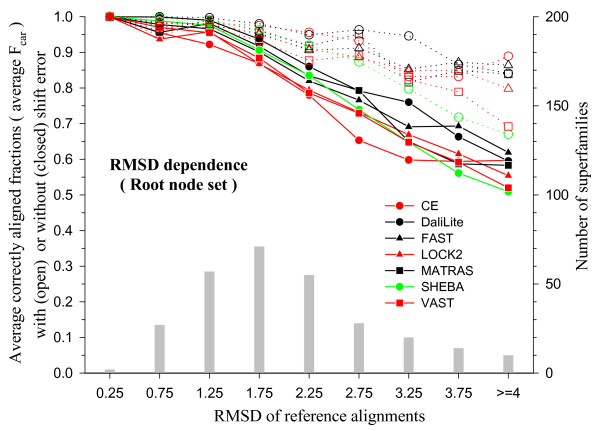
**RMSD dependence of F_car _in the root node set**. The structure pairs were superposed using the reference alignments to calculate the RMSDs. The test alignments were grouped into RMSD bins of size 0.5 Å and then the alignments within each bin were grouped according to its CD name for averaging. The avearge F_car _values are shown with the scale on the left y-axis: open symbols, F_car_(8); closed symbols, F_car_(0). The x-axis shows the midpoint of each RMSD bin. All the structure pairs with RMSD greater than 4.0 Å were collected in the last bin. The histogram (grey bars) shows the number of superfamilies in each bin with the scale on the right y-axis.

The dependence of the average F_car _values on structural similarity is shown in Figure 7, where the degree of structural dissimilarity is measured by means of the RMSD. This is the root-mean-square of the distances between the C_*α *_atoms of the residues aligned and superposed according to the reference alignment. As expected, average F_car _decreases as RMSD increases and F_car_(0) decreases more sharply than F_car_(8). Even the best performing method correctly aligns only about 80% of the residues without shift error, on average, as the RMSD approaches 3 Å, while the same method correctly aligns as much as 95% of the residues at this RMSD range if shift error is allowed.

**Figure 8:**
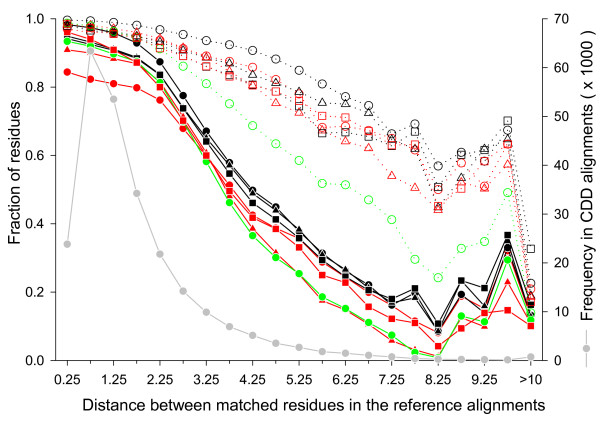
**Distance dependence of correctly aligned residue fractions**. The structure pairs were superposed using the reference alignments to calculate the distance between the C_*α *_atoms of all aligned residue pairs. The residue pairs were then grouped into distance bins of size 0.5 Å. The residue pairs that were 10 Å or more apart were collected in the last bin. For each test alignment, the fraction of correctly aligned residues were calculated (scale on the left y-axis) in each distance bin with (dotted lines with open symbols) or without (solid lines with closed symbols) shift error. The symbols and colors for the methods are the same as in the Figure 7. The solid grey circles and grey lines give the total number of residue pairs in each bin (scale on the right y-axis).

Since RMSD values can be heavily influenced by a small number of long distance pairs in the superimposed structures, we also measured the fraction of correctly aligned residues within each range of the C_*α *_distance between aligned residue pairs in the reference alignment (Figure 8). The distances are mostly less than 3.0 Å, but there are still a significant number of residue pairs that are more than 3.0 Å apart. The figure shows that correctly aligned residue fraction without shift error sharply decreases as the distance increases beyond 2.0 Å while the equivalent fraction with shift error decreases rather slowly. Thus, while there are relatively few residues aligned at distances larger than 2 or 3 Å, a large fraction of these residues are misaligned.

**Figure 9:**
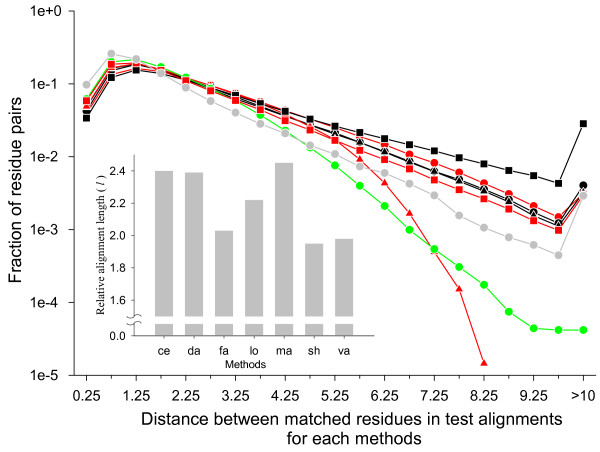
**Distance distribution of aligned residue pairs**. The structure pairs were superposed according to a given test or reference alignment and the distances between the C_*α *_atoms of aligned residue pairs were calculated. The residue pairs were then grouped into into distance bins of 0.5 Å size. Their relative frequency in each bin was plotted in logarithmic scale. The residue pairs 10 Å or more apart were collected in the last bin. The symbols and colors for methods are the same as in the Figure 7. The results from the reference alignment are shown by grey circles and lines. The inset shows the average alignment length of each method relative to that of the CDD alignment, where the method names on the x-axis were abbreviated by first two letters.

Figure 8 also shows that large fractions of the long distance pairs aligned in the reference alignments are not aligned or aligned with shift error of more than 8 residues in the test alignments. This could be observed if all automatic methods tended to align less number of long distance pairs compared to CDD. The distributions of the distances between aligned residue pairs in the test and reference alignments are shown in Figure 9. It shows that most structural alignment programs in fact produce more long distance aligned pairs than the reference alignment. LOCK2 and SHEBA are exceptions, which produce less aligned pairs with distances greater than 3.5 Å and 4.5 Å, respectively. Thus, most structure alignment programs do produce aligned pairs at long distances, but a large fraction of them are either wrong residue pairs or are grossly misaligned.

We note in passing an easily discernible feature on the length of the alignments that different structure alignment programs produce (inset of Figure 9). As expected, all programs produce longer alignments than the reference alignment, since CDD alignments are those of the conserved core regions in a set of multiple alignments whereas test alignments are pair-wise alignments that may include residues outside of the conserved core. But CE, DaliLite and MATRAS produce relatively long alignments on average, FAST, VAST and SHEBA produce relatively short alignments and LOCK2 is in between.

### Variations within and between superfamilies

The results described in the previous sections (except for Figures 8 and 9) were given in terms of the *f*_car _values averaged over all protein pairs and over all CDD superfamilies. However, each method gives alignment accuracies that vary greatly over different protein pairs and over different superfamilies.

**Figure 10:**
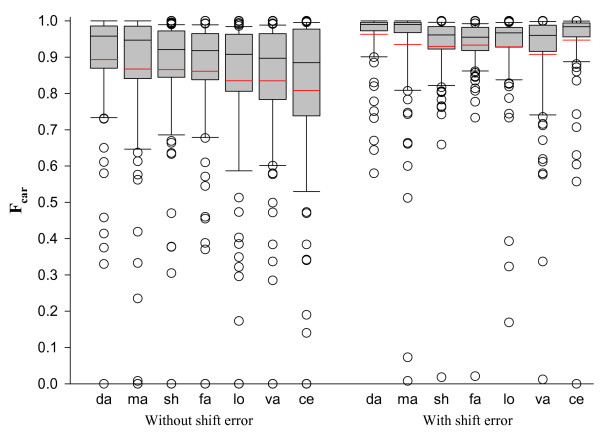
**The box plot of F_car _distributions for each method with (on the left panel) or without (on the right panel) shift error**. On the x-axis are the first two letters from each method name sorted according to the mean of F_car_(0). Each box plot shows the median (black line in the box), the 25th and 75th percentiles (box boundaries), and the 10th and 90th percentiles (error bars). The red line gives the average. The outliers outside of the 10th and 90th percentiles are shown as individual open circles.

The box plots in Figure 10 give the distribution of F_car_(0) and F_car_(8) values over the CDD superfamilies for each method. DaliLite has the narrowest distribution of F_car_(0) values with the highest mean and median while CE has the widest distribution with the lowest mean and median. All methods give F_car_(0) values less than 0.5 for a number of superfamilies and completely fail for at least one superfamily. The distribution for F_car_(8) is much tighter in comparison.

**Figure 11:**
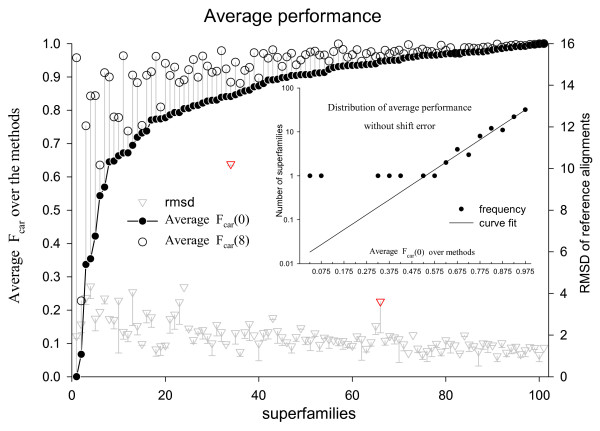
**The F_car _values averaged over the methods (left y-axis scale) and the average RMSD of the reference alignments for each superfamily**. The F_car_(0) (filled circle) and F_car_(8) (open circle) values (scale on the left y-axis) of each superfamily are connected by a vertical grey line. Average RMSDs are shown by inverse triangles with error bars on the negative side only (scale on the right y-axis). Two superfamilies with exceptionally high RMSD are marked in red. The structures in these superfamilies (one of the split cd00365 and cd00172) contain sub-structures that are flexibly joined to the rest of the structure. The inset shows the distribution of average F_car_(0) with bin size of 0.05 in semi-logarithmic scale. The dots represent the observed frequencies and the line is best fitting exponential curve to the observed frequencies.

**Figure 12:**
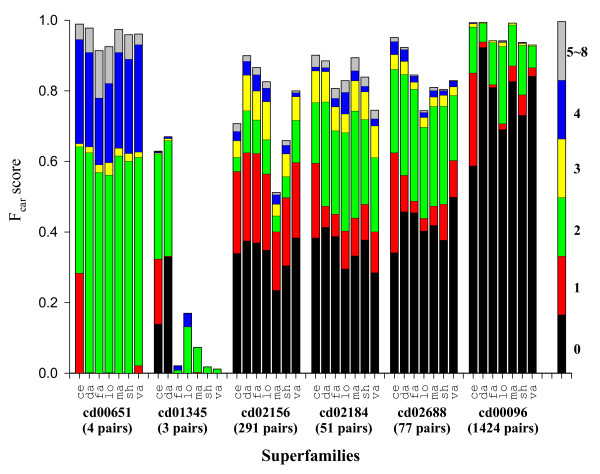
**Shift error profiles of the five outlier superfamilies from Figure 11 and the largest superfamily**. The name of the superfamily, and the number of the alignment pairs in it are shown at the bottom of the figure. The largest superfamily (cd00096, immunoglobulins) is included for reference as a "typical" superfamily. In each superfamily, seven methods are indicated by the first two letters of their names. Each bar is broken into segments whose length gives the fraction of the aligned residues with a given shift error, which is indicated in color according to the coloring scheme shown in the single bar on the right. Since most of the shift errors are at most 4 residues, the fractions having more than 4 residues were combined into one.

The existence of superfamilies for which different methods give zero F_car _value raises the possibility of systematic deviation of the result from human curation for some superfamilies. In order to identify such superfamilies, averages of F_car _values were calculated over all methods for each superfamily. Figure 11 shows the method-averaged F_car_(0) and F_car_(8) values for superfamilies sorted in the order of increasing F_car_(0) value. The distribution of the method-averaged F_car_(0) values over the superfamilies follows exponential decay except for five superfamilies with the lowest method-averaged F_car_(0) values (see inset of Figure 11). These superfamilies are listed in Table 2. All the methods give low F_car_(0) values for these five superfamilies (Figure 12).

**Table 2:** The largest CDD superfamily and the superfamilies for which all programs score poorly

Name	SCOP class	Pairs^‡^	Sub-families^§^	Description in CDD
cd00651	*α*+*β*	4	2	T-fold; Tunneling fold
cd01345	f^†^	3	3	OM_channels; Porin superfamily
cd02156	*α*/*β*	291	3	nt_trans; nucleotidyl transferase
cd02184	*α*+*β*	51	4	AroH_like;YgbB family
cd02688	all-*β*	77	8	E_set; E or "early" set of sugar utilizing enzymes
cd00096	all-*β*	1424	3	IG: Immunoglobulin domain family

^† ^Membrane and cell surface proteins and peptides

^‡ ^Number of pairs in the root node set in each superfamily

^§ ^Number of immediate subfamilies contributing structure pairs to the root node set

Included in Figure 11 are the RMSD values averaged for each superfamily. They generally decrease as the F_car_(0) value increases, although there are a couple of exceptions, as indicated by the red inverted triangles. None of the 5 superfamilies identified above has an exceptional RMSD value. This indicates that there is no gross error in the reference alignments for these superfamilies.

**Figure 13:**
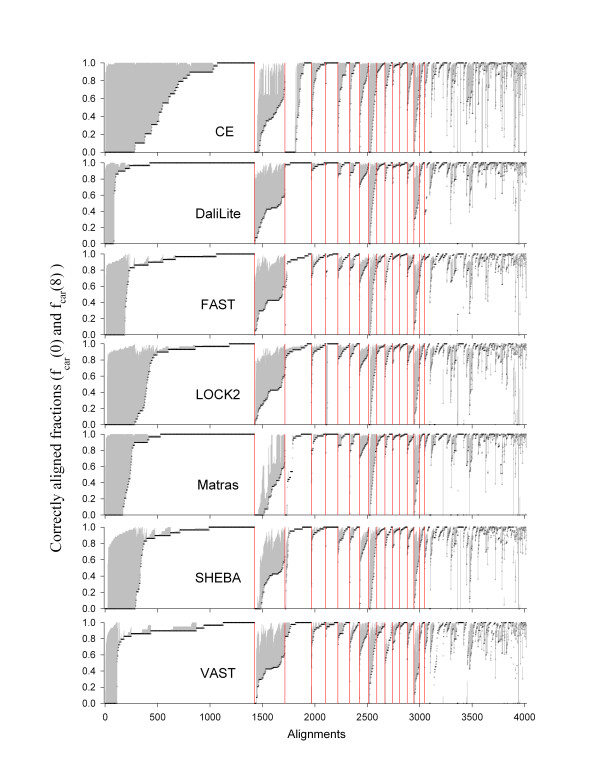
**The fraction of correctly aligned residues (*f*_*car*_) of each alignment and for each method**. The superfamilies along the x-axis were sorted in descending order of the number of alignments in each. The boundaries of those with 50 or more alignments are marked by red vertical lines. The alignments in each superfamily were sorted in ascending order of *f*_car_(0), which are shown in black circles. The grey vertical lines cover the range between *f*_car_(8) and *f*_car_(0) for each alignment. The methods are given in alphabetical order. Note that the order of superfamilies along the x-axis is preserved for all methods, but the order of the individual alignments within a superfamily is not since they are sorted by *f*_car_(0) values, which are specific for each method. Superfamilies marked by the red boundary bars are, from left: cd00096, **cd02156**, cd01983, cd00900, cd00657, cd02019, cd03440, cd01292, **cd02688**, cd00314, cd00196, cd00650b, cd00650a, cd00768, **cd02184**, and cd00267. The bold-faced superfamilies are three of the five exceptional ones identified in Figure 11 and listed in Table 2. These are those for which the *f*_car_(0) values are low (longest grey lines) for all methods.

Some members of these superfamilies were visually examined. The poorest results were obtained for the cd01345 superfamily. This superfamily consists of the outer membrane porins, which are large *β*-barrel structures. Two such barrels can be aligned in many different ways if sequence information is not used. For the four alignments in cd00651, all the methods produced similar alignments, where most of the residues were shifted by 2 residues in *β*-strands and/or by 4 residues in *α*-helices from the CDD alignments. Similarly shifted alignments were observed in up to 20% of the alignments in cd02688 (see Figure 13 for the detail). For the cd02156 and cd02184 superfamilies, the reference alignments look unusual in that some *β*-strands are out of phase or two residues off, according to our visual inspection of the structures superposed according to the CDD alignment (data not presented).

One notable feature is that CE produces more one-residue shifted alignments than other methods for 4 of the 5 superfamilies (red bars in Figure 12), as well as for cd00096 included here for reference as a typical superfamily.

In general, *f*_car _values also vary within each superfamily for all methods (Figure 13). Relatively large variation of *f*_car_(0) compared to *f*_car_(8) implies that there will be correspondingly large number of inconsistencies among the alignments of the superfamily members. For the largest superfamily, cd00096, all methods produced 5% (DaliLite) to 20% (CE) of alignments wherein all the residues are shifted. Some of these shifted alignments are as good as the reference alignments in terms of the RMSD and the number of aligned residue pairs, but are clearly wrong because the conserved cysteine residues that form the disulfide bond are not correctly aligned (See Figure 5 for an example). This kind of incorrect alignments in immunoglobulin were discussed by Gerstein and Levitt in the category of "hard to align" pairs [12].

### Architecture dependence of performance

It is known that some structure alignment programs show weakness in some specific architecture of the proteins in structure classification [7,28]. In order to examine possible such dependence in sequence alignments, the alignments were grouped by their SCOP class. The main four classes, *α*, *β*, *α*/*β *and *α*+*β*, were separately considered and the remainder were combined into the "others" class. For this study, we excluded the 5 outlier superfamilies of Figure 12.

**Figure 14:**
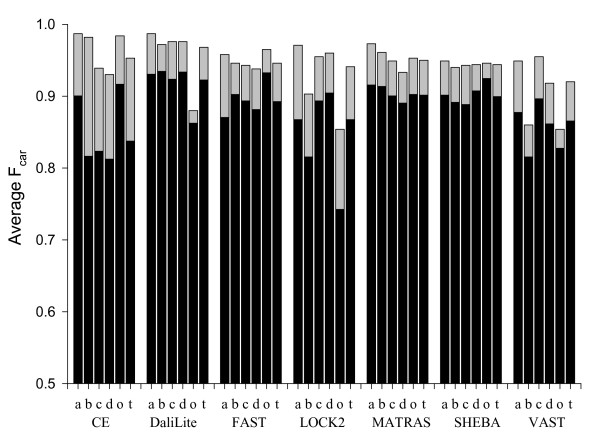
**Dependence of F_car _on the protein structural classes**. The F_car_(0) (solid bar) and F_car_(8) (full bar) values were grouped and averaged over each SCOP class. For this analysis the 5 extreme superfamilies from Figure 11 were not included. The symbols a, b, c, d, and t represent all-*α*, all-*β*, *α*/*β*, *α*+*β*, "others" and all classes, respectively. The method names are given on the x-axis.

Each method shows a different pattern of relative weaknesses for different SCOP classes (Figure 14). CE gives relatively poor results for *β*-sheet-containing structures (all-*β*, *α*/*β*, and *α*+*β *classes), DaliLite for "others" class, and LOCK2 and VAST for all-*β *and "others" classes. FAST, MATRAS, and SHEBA do not show such significant weakness in any particular class. Interestingly, secondary-structure-independent methods such as CE, FAST and SHEBA show good performance for the "others" class. Inclusion of the five outlier superfamilies gives substantially similar results (see supplementary material) except that the average F_car _is lower for the "others" class for all methods because of the cd01345 superfamily in this class.

## Discussion

### Performance difference of the methods

A significant observation in this study is that DaliLite produces the most accurate structure-based sequence alignment, while CE is clearly not as good when shift error is not allowed (Figure 2). This result contrasts with an earlier evaluation study[9] wherein DaliLite was found to produce worse alignments than CE in terms of geometric measures, which include RMSD. Our result is more consistent with Sierk and Pearson's work[7], in which DaliLite was found to be the best followed by MATRAS, although they measured classification ability rather than alignment accuracy, using CATH database as the gold standard.

DaliLite, MATRAS and FAST, which are relatively good performers in our analysis, are based on the comparison of intra-molecular distance matrices without resorting to rigid body rotation during structural alignment [26,29]. Thus, structural superposition is not necessary to obtain a good sequence alignment. Also, different algorithms give different performances depending on how much shift error is allowed and on the secondary structure content of the structure. DaliLite, LOCK2 and VAST probably depend more on secondary structures than other programs and perform less well for "others" class of structures. CE tends to give inaccurate alignments for *β*-containing structures but performs well when some shift error is allowed, which makes it more suitable for homology detection and structure classification tasks. CE, DaliLite, and MATRAS produce long alignments (inset of Figure 9). MATRAS produces longer alignments on average than DaliLite, but performs less well.

Such differences among the methods were not observed with the terminal node set (Figure 2). FAST was evaluated by its own authors using the overlap score, which is the same as *f*_car_(0), and HOMSTRAD as the gold standard[26]. The reported accuracy of 96% is consistent with our observation using the terminal node set. This suggests that the sequence similarity of the proteins in the HOMSTRAD dataset is perhaps similar to that of our terminal node set, which is made of "easy" cases for which all methods perform similarly well. The present study shows the advantage of using the root node set for evaluation since it has a higher discrimination power than the terminal node set (Figure 2).

### Alignment accuracy measures

We used *f*_car_(0) and *f*_car_(8) values almost exclusively as the measures of accuracy of alignments. These are the fraction of residues that are correctly aligned within the specified alignment shift error. As mentioned above, *f*_car_(0) values are the suitable measures when accurate alignment is essential as in building profiles. On the other hand, for the purposes of finding structurally similar proteins and for the structure classification, *f*_car_(8) may be a better measure to use. Measures such as *f*_car_(8) is probably preferable over a quantity that measures how well the program reproduces an existing structure classification dataset such as SCOP or CATH; the latter test brings in a set of issues, such as the human classification versus machine comparison and the effect of clustering [[28] and manuscript in preparation], which are only peripherally related to the performance of the pair-wise structure alignment program itself.

The *f*_car _measures can be used only when one has a reliable set of alignments that can be considered to be true. We used the NCBI's CDD alignments for this purpose. When such standard is not available, one has to use some absolute measure of the goodness of the alignments. Authors of SHEBA, for example, which include one of us (BL), used the number of residue pairs aligned within a given distance as the measure of goodness. Kolodny et al. [9] define four different measures, each of which is some combination of the number of aligned residues and the RMSD. As mentioned above, use of these measures results in a different ranking of the programs. It is easy to understand why the RMSD is included in the goodness measure that is basically based on how many residues a program aligns; the alignment length can be increased arbitrarily until it encompasses the whole protein if RMSD is not considered. However, as can be seen in Figures 8 and 9, our reference alignments include a significant number of conserved core residue pairs that are rather far apart. Simply discouraging the alignment of such pairs is not necessarily the desired characteristic of a good structure alignment program and it may not be easy to find the proper combination of the number of aligned residues and the RMSD that will correctly assess the accuracy of a structure alignment program.

### CDD as reference alignments

There are advantages to using the alignments from CDD as the reference dataset since they are human-curated and include sequences of both high and low sequence similarities. Although VAST alignment results are consulted by the NCBI curators of CDD, there does not seem to be a VAST-specific bias since VAST does not perform particularly well among the tested methods (Figure 2).

An obvious drawback is that CDD gives alignments of only the conserved core region from multiple alignments. A pairwise alignment will generally align more residues outside of the conserved core, but the accuracy of these alignments cannot be assessed using this reference set of alignments. Our assumptions are that any good alignment program should do well for the conserved core residues and that a program that aligns the conserved core residues well will also align the non-core residues better than other programs.

### Imperfectness of alignments

Although we investigated only the conserved core regions of the alignments, it is clear that all structure alignment programs often produce alignments with all or part of this core region of the structures misaligned (See Figures 4 and 5). The correctly aligned fraction never reaches 95% even after shift error is allowed for up to 8 residues (Figures 2, 3 and 13) and it decreases rapidly as the sequence similarity decreases or as the RMSD increases (Figures 6, 7 and 8).

A possible reason for such discrepancy is the potential errors in the human-curated reference alignments. It was pointed out in the Results section that some of the CDD alignments were unusual from the point of view of purely structural alignment. However, we believe that this is not the major contributor to the observed discrepancy according to two limited investigations we made as described below.

If the problem is in the reference alignment, all methods are likely to score poorly. But, as shown in Figure 11, there are only 5 superfamilies that are exceptionally poorly aligned by all methods and inclusion or exclusion of these superfamilies had little effect on the overall alignment accuracies.

**Figure 15:**
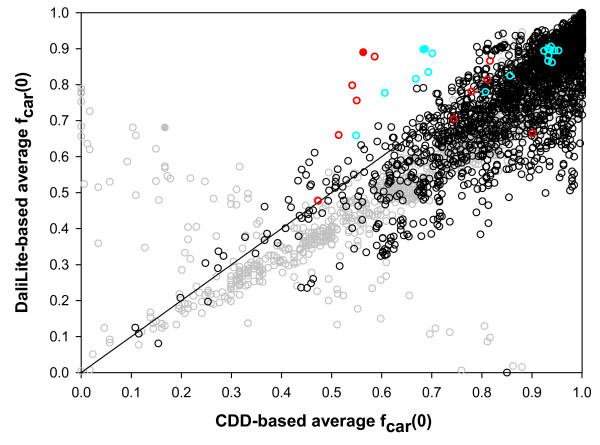
**Correlation between CDD-based and DaliLite-based *f*_car_(0)s**. The 3581 pairs of reference alignments from the root node set were considered, excluding those from the five outlier superfamilies (Figure 11) and additional 10 pairs for which DaliLite didn't produce sequence alignment at all. The x- and y-axes give *f*_car_(0) values averaged over the six methods, excluding DaliLite, with CDD and DaliLite alignments as references, respectively. The pairs from the three superfamilies, cd01984, cd00531 and cd00096, are colored red, cyan and grey, respectively. The remainder are colored black. Filled circles indicate the pairs visually examined, whose alignments are shown in Figures 5 and 16.

**Figure 16:**
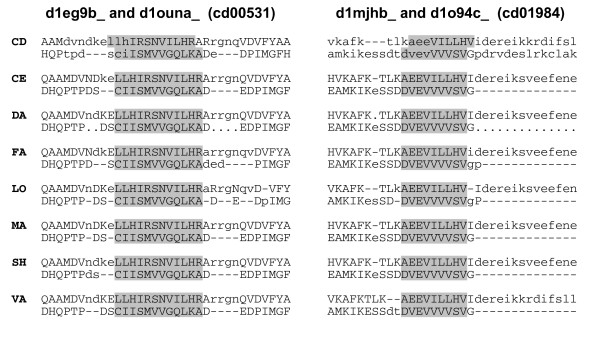
**Two examples of alignments on which all methods agree but which is different from that of CDD**. The alignments on the left and right panels are from cd00531 and cd01984, respectively. The names of the protein domains aligned are given at the top of each panel. The method which generated the alignment is indicated at the left of the sequence by the first two letters of its name. Only a part of the sequence is shown in each case, which includes the region that is aligned differently by the methods and by the CDD. The aligned residues are indicated by uppercase letters. The residues aligned identically by all methods but differently by CDD are shaded.

Figure 15 shows the results of another test we made. It shows method-averaged *f*_car_(0)s for all test alignments, except the five superfamilies mentioned above, using the CDD alignments as the reference alignment (x-axis) and the DaliLite alignment as the reference alignments (y-axis). If the CDD and DaliLite alignments were the same, all points would fall on the diagonal line in the figure. Since CDD aligns only the core region while other methods align non-core residues also, the *f*_car_(0) values are expected to be larger when the CDD is used as the reference than when DaliLite is used. Figure 15 shows that most points (93%) indeed fall below the diagonal. The points that lie above the diagonal in Figure 15 represent the pairs for which the methods, on average, agree better with DaliLite than with CDD. If CDD alignment is in error for a pair, the corresponding point is likely to be found among these points above the diagonal. One can see that there are relatively few points above the diagonal. We have visually inspected the structural superposition for a few of these points. Many points were for immunoglobulin pairs (cd00096), which were aligned correctly by CDD, but many or all automatic programs made one pitch shifted alignment of the type shown in Figure 5. Most of the other points that are far above the diagonal are for pairs in two superfamilies, cd00531 and cd01984, (red and cyan points in Figure 15, respectively). For some of these pairs, all or most of the methods agreed on an alignment, which was different from the CDD alignment, at one part of the structure. Figure 16 shows such alignments for two pairs colored solid in Figure 15. In both cases, inspection of the multiply superposed structures indicates that the alignment from the automatic programs is clearly superior to the CDD alignment. Thus, we could identify some CDD alignments that appear to be in error, but these cases are few in number.

A related possibility is that there are equally good alternate alignments for many of the structure pairs, as was pointed out by many authors [4,5,10-12]. The alternate alignments can affect the whole structure or only a part of the structure. The possibility of such alternates will increase for evolutionarily distant pairs as the sequence similarity becomes low and the structures acquire distinct differences. The fact that residue pairs that are more than 3.0 Å apart in the reference alignment are heavily misaligned in the test alignments (Figure 8) suggests that this could be a significant contributor to the overall discrepancy between the test and reference alignments. In such circumstances, even structure-based sequence alignment can benefit from multiple alignments and from including the evolutionary relation between sequences.

A third possibility is of course the imperfection of the pair-wise structure alignment programs. The fact that different programs behave differently for the same set of data indicates that they are not yet perfect. We have observed that different programs totally fail for different sets of protein pairs. We have observed many instances wherein all or part of the structure is shifted by 2 or 4 residues compared to the reference alignment. In the example shown in Figure 5, the DaliLite alignment is clearly wrong because the cysteine residues do not align. We are also surprised by the large number of cases wherein the alignment is shifted by an odd number of residues for all or part of the structure. It is definitely our impression that there is room for improvement in the structure alignment programs.

## Conclusion

The accuracy of the sequence alignments produced by 7 commonly used structure alignment programs was evaluated using the sequence alignments from NCBI's human-curated Conserved Domain database as the standard of truth and the "correctly" aligned fraction of residues as the alignment quality measure. These programs mis-align 11–19% of the conserved core residues on average for structure pairs in the same CDD root node but not in the same child node. DaliLite gave the best results among the programs tested. The alignment quality varied depending on the program used, on the protein structural type (SCOP Classes), and on the degree of sequence and structural similarity.

## Methods

### Reference alignment sets

Since CDD includes hundreds of families imported directly from outside sources, such as Pfam, COGs and SMART, we collected only the expert-curated CD (Conserved Domain) families, whose names always begin with "cd" [21]. There were 2,009 such CDs (CDD v.2.07 as of 04/04/2006) organized in a hierarchical manner: 285 singleton CDs (without children or parents), 146 CDs from root nodes, 1,440 CDs from terminal nodes, and 138 CDs from internal nodes (between root and terminal nodes in CD hierarchy). We selected 828 CDs with at least two 3D structures and, using *cddalignview *from the NCBI c++ toolkit, extracted multiple sequence alignments from their ".acd" files. This subset includes 220 singletons, 135 root nodes, 367 terminal nodes and 106 internal nodes. Total 21,140 pairwise alignments were prepared from these multiple alignments. Each sequence in the alignments included all the unaligned residues at both termini, since -*lefttails *and -*righttails *options were used with *cddalignview*.

CDD uses curated domains based on MMDB [30-32]. For this study, we adopted the ASTRAL SCOP domains (ASTRAL SCOP 1.69) because they were better documented. The ASTRAL domain sequences and structures were downloaded from ASTRAL web site [33]. Finding the ASTRAL domain corresponding to a CDD domain, however, is not trivial, because domain definitions do not always coincide. In order to determine which ASTRAL domain is associated with which CDD domain, we used a sequence alignment procedure (Lobster package[34]). First, each sequence in a given CDD alignment was aligned to all the ASTRAL domain sequences derived from the same PDB structure. An ASTRAL domain was selected if at least 70% of its residues were covered by the CDD aligned span. A CDD aligned span is the sequence segment spanned by the first and the last aligned residues in the CDD alignment. This means that a CDD sequence can correspond to more than one ASTRAL domain. When this happened, all the domains were kept, which meant that the single CDD domain was effectively split into more than one domain according to ASTRAL SCOP definition. If an ASTRAL domain was not assigned to a sequence of a CDD aligned sequence pair, the pair was omitted. We also required that the aligned region between the domain spans include at least 20 residue pairs and cover at least 70% of the shorter span. A domain span here is defined for each ASTRAL domain as the region from the first to the last aligned residues within the boundaries of the domain. Its length is the number of the residues and gaps in the span. After this procedure, the dataset contained 6,425 pairwise alignments from the root nodes, 2,351 from the internal nodes, 2,809 from the terminal nodes, and 2,979 from the singletons. Each reference alignment is associated with a pair of ASTRAL domains and the pair-wise CDD sequence alignment.

We used only the root and terminal node sets. In order to select alignments specific to the root node set, the alignments were excluded from the root node set if their domain pair was also included in the internal or terminal node set. The pairs with 80% or more sequence identity (among aligned residue pairs) were also removed from both the root and the terminal node sets. If a structure in the aligned pair did not contain the side chains or was derived by NMR, the pair was also eliminated. The final reference alignment sets consisted of 2,199 alignment pairs for the terminal node set and 4,017 pairs for the root node set (Additional file 3).

### Structure alignment programs

For various reasons, we could not evaluate all known structure alignment programs. We selected programs mainly based on their availability. Some programs were difficult to use because they failed for some of the structure pairs for unknown reasons or generated sequence alignments that were different from what were implied by other measures such as RMSD values. Finally we included CE (Algorithm 1.0, Alignment calculator 1.02)[35], DaliLite_2.4.1 [29], LOCK2 [36], FAST [26], MATRAS (version 1.2)[37], VAST (directly from Dr. Gibrat) [31] and SHEBA-4.0 [38]. SSEARCH from FASTA3 package [27] was used for pure sequence alignment. The MATRAS and VAST were kindly given to us by the authors; others were downloaded from their websites.

Each program was run with its default setting. CE needs SEQRES sequence to recognize the residues as they are in the PDB file. Since such information is not included in PDB-style ASTRAL domain files, the three-letter symbols were derived from the ATOM records in the PDB-style files. When the secondary structure information is explicitly required, DSSP[39] was used. VAST includes companion programs, which derive the secondary structures and SCOP domains from the original PDB files containing the whole structure. When a program generates more than one alignment for a given structure pair as in DaliLite and VAST, the first alignment in the output file was chosen for the evaluation.

### Sequence alignment quality measure

A test alignment was generated for each reference alignment by running the structure alignment program on the two ASTRAL domains assigned to the reference alignment. The test alignment generated then need to be compared to the reference alignment for quality assessment. However, the protein sequence in the test alignment is often not identical to that in the reference alignment. For example, residues missing in the crystal structure do not appear in the test alignment. Some non-standard amino acids are simply removed (FAST) or marked with the extended amino acid symbols (LOCK2) – B, Z or X. Also, CE removes unaligned N-terminal and C-terminal residues. These and other sequence related issues involved in comparing different sequence alignments have been addressed before [4]. In this study, we used a sequence alignment procedure (see below) in order to establish the correspondence between residues in the test and the reference alignment sequences. In principle, there are cases when an unambiguous correspondence cannot be made even by the sequence alignment. For instance, if there are tandem repeats in the sequence and one of these contains a gap, the gap can be relocated without cost by the sequence alignment procedure. Fortunately, we have not detected such ambiguity in the aligned regions of any of our reference alignments.

We used the C++ class library included in the Lobster package to handle sequence alignments [34]. Two sequences derived from the same protein, one from the test and the other from the reference alignments, were aligned. The lengths and the one-letter symbols of these sequences can be different even though both are for the same protein. Then the serial numbers of the residues in the reference alignment sequence were assigned to the residues in the test alignment sequence. After this step, residues were identified by means of the assigned serial numbers alone, so that different symbols for the same residue were allowed. Also, the residues in the reference alignment sequence that do not appear in the test alignment sequence, either because the residue is missing in the crystal structure or because the ASTRAL domain spans less than the whole reference aligned span, are marked as unaligned in the reference sequence and not considered further.

**Figure 17:**
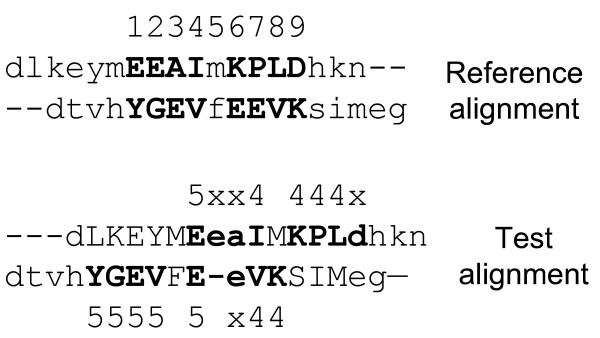
**The concept of shift error and the fraction of correctly aligned residues (*f*_*car*_)**. Aligned and unaligned residues are indicated by uppercase and lowercase letters, respectively. Residues aligned in the reference alignment are in bold. The numbers above the reference alignment are the serial numbers of residues in the aligned span of the reference alignment. The shift errors (*δ*) are shown on the test alignment, where 'x' indicates that *δ *is not defined for the unaligned residue in the test alignment although it is aligned in the reference alignment. The two residues of the pair #5 are not considered at all for the shift error calculation since they are not aligned in the reference alignment. For this example, *f*_car_(0) = 0/16, *f*_car_(4) = 6/16, and *f*_car_(8) = 12/16.

For a residue R in sequence A that is aligned in both the reference and test alignments, its shift error, *δ*, is defined as the difference in the serial number of the two residues of sequence B that are aligned to R in the reference and test alignments (Figure 17). We consider only the residues that are aligned in the reference alignment. If a residue aligned in the reference alignment is not aligned in the test alignment, the shift error was not defined (Figure 17).

For each structure pair, let *r *and *t *be the number of aligned residue pairs in the reference and test alignments, respectively, and let *m*(*δ*) be the number of aligned residues in both sequences with shift error up to *δ*. We define the fraction of "correctly" aligned residues, *f*_*car*_(*δ*), and the relative alignment length, *l*, as  and . The *f*_*car*_(*δ *= 0) is the same as *f*_D_, which Sauder et al. [4] called the "developer's viewpoint" score. This has also been called the sensitivity of sequence alignment [40,41].

## Abbreviations

### Program names

CE, **C**ombinatorial **E**xtension; DaliLite, standalone version of DALI (**D**istance m**A**trix **ALI**gnment); DSSP, **D**efinition of **S**econdary **S**tructure of **P**roteins given a set of 3D coordinates; FAST, Recursive acronym for **F**AST **A**lignment and **S**earch **T**ool; FASTA3, DNA and Protein sequence alignment software package; LOCK2, Improvements over LOCK (Hierarchical protein structure superposition); MATRAS, **MA**rkovian **TRA**nsition of protein **S**tructure; SHEBA, **S**tructural **H**omology by **E**nvironment-**B**ased **A**lignment; SSEARCH, Smith-Waterman search; VAST, **V**ector **A**lignment **S**earch **T**ool.

### Database names

ASTRAL, compendium for protein structure and sequence analysis; BaliBase, **B**enchmark **Ali**gnment data**Base**; CATH, Hierarchical classification of protein domain structures, which clusters proteins at four major levels, Class (**C**), Architecture (**A**), Topology (**T**) and Homologous superfamily (**H**); CAMPASS, **CAM**bridge database of **P**rotein **A**lignments organised as **S**tructural **S**uperfamilies; COGs, **C**lusters of **O**rthologous **G**roups of proteins; DBAli, **D**ata**B**ase of structure **Ali**gnments; HOMSTRAD, **HOM**ologous **STR**ucture **A**lignment **D**atabase; MMDB, **M**olecular **M**odelling **D**ata**B**ase; OXBench, benchmark for evaluation of protein multiple sequence alignment accuracy; PALI, database of **P**hylogeny and **ALI**gnment of homologous protein structures; PASS2, **P**rotein **A**lignments organised as **S**tructural **S**uperfamilies (version **2**); Pfam, multiple sequence alignments and HMM-profiles of protein domains; PDB, **P**rotein **D**ata **B**ank; PREFAB, **P**rotein **REF**erence **A**lignment **B**enchmark; S4, **S**tructure-based **S**equence alignments of **S**COP **S**uperfamilies; SABmark, **S**equence **A**lignment **B**enchmark; SCOP, **S**tructural **C**lassification of **P**roteins; SMART, **S**imple **M**odular **A**rchitecture **R**esearch **T**ool; SUPFAM, database of potential protein **SUP**er**FAM**ily relationships.

### Alignment quality measure

GSAS, **G**apped **SAS**; SAS, **S**tructural **A**lignment **S**core.

### General

RMSD, Root-mean-square of C_*α *_distances between aligned pairs after structural superposition; NCBI, **N**ational **C**enter for **B**iotechnology **I**nformation; CD, Conserved Domain in CDD; CDD, NCBI's Conserved Domain Database.

## Competing interests

The author(s) declares that there are no competing interests.

## Authors' contributions

BL generated the original idea, CK executed the research, and both CK and BL wrote the paper. Both authors read and approved the final manuscript.

## Supplementary Material

Additional file 1 The results for the terminal node set and SCOP class dependency including outlier superfamilies. The A, B, C and D are the counterparts of Figures 4, 6, 8 and 14, respectively. Click here for file

Additional file 2 The number of times when a method was the best or in the best class or when it failed. This is complementary to Figure 3, excluding the five outlier superfamilies from Figure 11. Click here for file

Additional file 3 Root node data set. The sequence alignments used in this study, including those generated by different methods and derived from CDD. Click here for file
