# Supplementary figures and images for: Accuracy of structure-based sequence alignment of automatic methods
Source: BMC Bioinformatics. 2007 Sep 20;8:355. doi: 10.1186/1471-2105-8-355 (PMC2039753; doi:10.1186/1471-2105-8-355)

**A**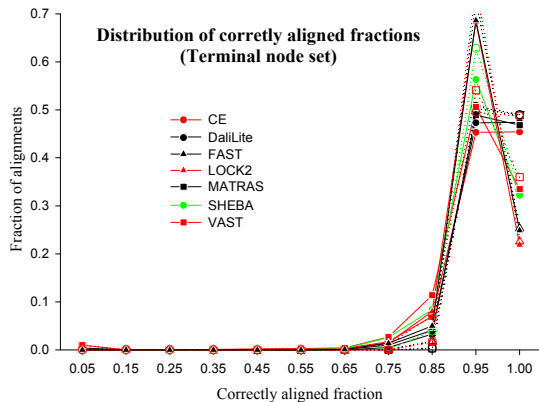**B**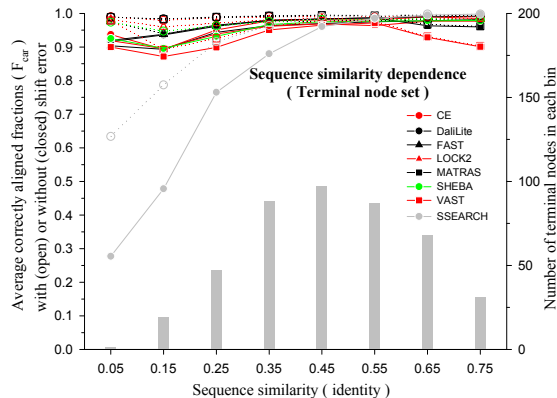**C**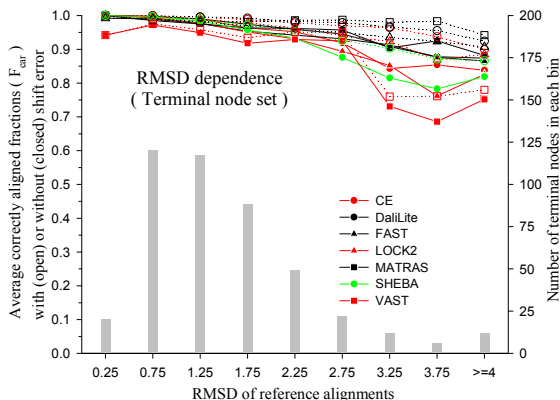**D**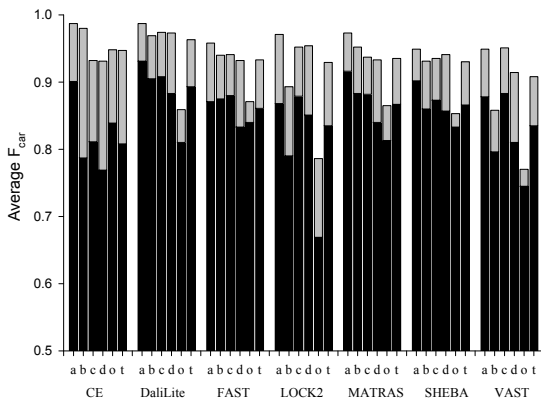

Supplement: Additional file 1 — The results for the terminal node set and SCOP class dependency including outlier superfamilies. The A, B, C and D are the counterparts of Figures 4, 6, 8 and 14, respectively. [file 1471-2105-8-355-S1.pdf]
