# Supplementary material for: Accuracy of structure-based sequence alignment of automatic methods
Source: BMC Bioinformatics. 2007 Sep 20;8:355. doi: 10.1186/1471-2105-8-355 (PMC2039753; doi:10.1186/1471-2105-8-355)
Supplement: Additional file 2 — The number of times when a method was the best or in the best class or when it failed. This is complementary to Figure 3, excluding the five outlier superfamilies from Figure 11. [file 1471-2105-8-355-S2.pdf]

The number of times when a method was the best or in the best class or when it failed[a]

f(0) including immunoglobulins				f(8) including immunoglobulins			
Method[b]	Unique top[c]	Top Class[d]	Failure[e]	Method	Unique top	Top Class	Failure
ce	169	1869	828	ce	*282*	3354	152
da	*440*	*3348*	*126*	da	187	*3542*	*27*
fa	123	2733	252	fa	18	3057	32
lo	51	2641	488	lo	5	3316	47
ma	283	2966	346	ma	258	3399	94
sn	81	2658	414	sn	27	3067	62
va	120	2179	201	va	139	2795	73
**f(0) without immunoglobulins**				**f(8) without immunoglobulins**			
ce	157	1435	282	ce	*239*	1944	149
da	*234*	*2048*	*41*	da	172	*2119*	27
fa	96	1805	48	fa	12	1935	*23*
lo	32	1768	91	lo	5	2066	28
ma	194	1884	108	ma	201	1989	90
sn	65	1798	79	sn	26	1892	36
va	99	1536	87	va	137	1834	68

[a]The best number in each category is italicized. All methods achieved less than 0.5 of $f_{car}(0)$, for 15 pairs from cd00096 and 2 pairs from cd00105. For only one pair from cd00105, $f_{car}(8)$ values of all methods are less than 0.5, probably because one of the structure has relatively large fraction of coordinate-missing residues and the structures are small. The five outlier superfamilies from Figure 11 were excluded, leaving 3591 pairs in total.

[b]The method is indicated by the first two letters of its name.

[c]The score of the second best method is lower than that of the best one by at least 0.1

[d]The score difference of each method from the best one is 0.1 or less.

[e]The score of the method is 0.5 or less.
